# B-Cell Receptor-Associated Protein 31 Promotes Metastasis *via* AKT/β-Catenin/Snail Pathway in Hepatocellular Carcinoma

**DOI:** 10.3389/fmolb.2021.656151

**Published:** 2021-06-11

**Authors:** Tengfei Liu, Junming Yu, Chao Ge, Fangyu Zhao, Chunxiao Miao, Wenjiao Jin, Yang Su, Qin Geng, Taoyang Chen, Haiyang Xie, Ying Cui, Ming Yao, Jinjun Li, Helei Hou, Hong Li

**Affiliations:** ^1^State Key Laboratory of Oncogenes and Related Genes, Shanghai Cancer Institute, Renji Hospital, Shanghai Jiao Tong University School of Medicine, Shanghai, China; ^2^Qi Dong Liver Cancer Institute, Qi Dong, China; ^3^Department of General Surgery, The First Affiliated Hospital, School of Medicine, Zhejiang University, Hangzhou, China; ^4^Cancer Institute of Guangxi, Nanning, China; ^5^Precision Medicine Center of Oncology, The Affiliated Hospital of Qingdao University, Qingdao, China

**Keywords:** BAP31, hepatocellular carcinoma, metastasis, AKT/β-catenin, Snail, heterogeneity

## Abstract

Hepatocellular carcinoma (HCC) is one of the most lethal cancer worldwide, characterized with high heterogeneity and inclination to metastasize. Emerging evidence suggests that BAP31 gets involved in cancer progression with different kinds. It still remains unknown whether and how BAP31 plays a role in HCC metastasis. Epithelial–mesenchymal transition (EMT) has been a common feature in tumor micro-environment, whose inducer TGF-β increased BAP31 expression in this research. Elevated expression of BAP31 was positively correlated with tumor size, vascular invasion and poor prognosis in human HCC. Ectopic expression of BAP31 promoted cell migration and invasion while BAP31 knockdown markedly attenuated metastatic potential in HCC cells and mice orthotopic xenografts. BAP31 induced EMT process, and enhanced the expression level of EMT-related factor Snail and decreased contents and membrane distribution of E-cadherin. BAP31 also activated AKT/β-catenin pathway, which mediated its promotional effects on HCC metastasis. AKT inhibitor further counteracted the activated AKT/β-catenin/Snail upon BAP31 over-expression. Moreover, silencing Snail in BAP31-overexpressed cells impaired enhanced migratory and invasive abilities of HCC cells. In HCC tissues, BAP31 expression was positively associated with Snail. In conclusion, BAP31 promotes HCC metastasis by activating AKT/β-catenin/Snail pathway. Thus, our study implicates BAP31 as potential prognostic biomarker, and provides valuable information for HCC prognosis and treatment.

## Introduction

Hepatocellular carcinoma (HCC) is one of the leading causes in cancer-related death, mainly caused by HBV and HCV infection, aflatoxin exposure and alcoholism (Chen et al., 2016; Siegel et al., 2020). Characterized with a vast molecular heterogeneity, HCC develops in a sequential evolution from dysplastic lesions to advanced stages (Marquardt et al., 2015), accompanying with cellular phenotype switch to more aggressive state and subsequent metastasis (Giannelli et al., 2016). Therefore, it is vital to seek for effective and sensitive factors for the monitor and treatment of HCC to improve the patient prognosis.

BAP31 (B-cell receptor associated protein 31) is encoded by *BCAP31*, which is located on chromosome X. The encoded protein is a transmembrane protein of endoplasmic reticulum, which gets involved in the transport of membrane proteins from endoplasmic reticulum (Annaert et al., 1997) and caspase 8-mediated apoptosis (Ng et al., 1997; Ng and Shore, 1998). In recent years, it has arisen increasing attention concerning its role in tumor development. In cervical cancer, BAP31 may act as a predictive factor and contribute to tumor progression by influencing cell cycle, apoptosis and cytoskeleton assembly (Dang et al., 2018; Wang et al., 2019). Xu et al. (2019) has reported that MiR-451a/BAP31 axis can inhibit cell proliferation and increase apoptosis *via* inducing ER stress in colorectal cancer. As cancer-testis antigen, BAP31 DNA vaccines exert potent anti-tumor activity in therapeutic model using B16 melanoma cells (Yu et al., 2015). One recent study has investigated that BAP31 may promote HCC cell proliferation by stabilizing SERPINE2 (Zhang et al., 2020). However, the role of BAP31 in HCC metastasis remains poorly understood.

Malignant progression of HCC has exhibited complex heterogeneity, involving altered plasticity of epithelial hepatocytes, which is commonly recognized as epithelial-mesenchymal transition process (EMT) (Giannelli et al., 2016). EMT is a multi-step biological process that drives epithelial cells to mesenchymal phenotype, such as losing cell-cell contacts due to ablation of epithelial marker E-cadherin (*CDH1*) and increasing motility marked with enhanced factors like Snail (*SNAIL1*) and so on (Peinado et al., 2007; Kudo-Saito et al., 2009). As a hot spot in cancer research, EMT gets interacted and regulated by multiple signaling pathways, including PI3K-AKT, which is involved in a variety of process such as tumor proliferation, metastasis, metabolic reprogramming, etc. (Liu et al., 2014; Xie et al., 2017; Rebouissou and Nault, 2020). Moreover, AKT/β-catenin activation also holds a lot in EMT induction and has drawn intense interest in cancer research (Zhao et al., 2017; Yuan et al., 2020).

As a classical inducer for EMT (Dituri et al., 2019), transforming growth factor-β (TGF-β) inclines to increase BAP31 expression in this study. It is elucidated that BAP31 expression is increased in HCC tissues and high BAP31 level predicts shorter survival time. Furthermore, we have demonstrated that BAP31 can activate AKT/β-catenin/Snail pathway, which facilitates HCC metastasis *in vitro* and *in vivo*. This study strongly highlights the significance of BAP31 in the progression of HCC and therefore provides a potential target in liver cancer therapy.

## Materials and Methods

### Patient Samples and Immunohistochemistry

Immunohistochemistry (IHC) analysis was performed in a tissue microarray containing 236 Hepatocellular carcinoma tissues to detect BAP31 expression as previously described (Li et al., 2011). The graphic results were captured using Precipoint M8 (Precipoin, Freising, FB, Germany). The results of IHC were assessed by two independent investigators according to scores of staining intensity and percentage of positive cells. Staining intensity was scored by the following criteria: 0, negative; 1, weak; 2, moderate; 3, strong. The IHC results were created from these two parameters as follows: no staining (intensity 0) was scored 0, intensity 1 in ≤ 70% of tumor cells or intensity 2 in ≤ 30% of tumor cells was scored 1, intensity 1 in >70% of tumor cells or intensity 2 in >30% but ≤70% of tumor cells or intensity 3 in ≤30% of tumor cells was scored 2, intensity 2 in >70% of tumor cells or intensity 3 in >30% but ≤70% of tumor cells was scored 3, and intensity 3 in >70% of tumor cells was scored 4. A score of 0–2 was considered to represent low expression and a score of 3–4 was considered to represent high expression. None of the patients received any preoperative radiation or chemotherapy. The study was approved by the Research Ethics Committee of Renji Hospital, Shanghai Jiao Tong University School of Medicine, and informed consent was obtained from each patient from the Zhejiang University (Hangzhou, China) and the Qidong Liver Cancer Institute (Qidong, China), in accordance with the World Medical Association’s Declaration of Helsinki.

### Cell Lines and Reagents

HEK-293T (CRL-11268^TM^), PLC/PRF/5 (CRL-8024^TM^), Hep3B (HB-8064^TM^), SNU449 (CRL-2234^TM^), SNU387 (CRL-2237^TM^) and SNU398 (CRL-2233^TM^) cells were bought from American Type Culture Collection (Manassas, VA, United States); Huh7 (RCB1366) cell was purchased from the Riken Cell Bank (Tokyo, Japan); L-02 (GNHu6) and Li-7 (TCHu183) cells were obtained from the Cell Bank of Chinese Academy of Sciences (Shanghai, China); HCC-LY10 and HCC-LY5 cells were established from human primary hepatocellular carcinoma in our laboratory; MHCC-97, MHCC-97H, and MHCC-LM3 cell lines were purchased from Zhongshan hospital, Fudan university (Shanghai, China) (Li et al., 2001). All cell lines were cultured in Dulbecco’s modified Eagle’s medium (DMEM) containing 10% fetal bovine serum (FBS) at the condition of 37°C, 5% CO_2_. All cell lines were authenticated and characterized by Short Tandem Repeat (STR) profiles and were used less than 10 passages after authentication. TGF-β was purchased from R&D Systems (United States). The AKT inhibitor MK-2206 2HCI was provided by Selleck Chemicals (United States).

### Animal Experiments

1 × 10^6^ HCC cells stably expressing shBAP31 or NC were suspended in 40 μl of a mixture with serum-free DMEM/Matrigel (1:1 volume) for each 6- to 8-week-old male BALB/c nude mouse and orthotopically injected into the left hepatic lobe. After 6 weeks, all animals were sacrificed. The liver and lung tissues were excised and fixed in 10% neutral phosphate-buffered formalin for 72 h. H&E (hematoxylin & eosin) staining was performed to detect metastases. All mice received human care and were raised in SPF (Specific Pathogen Free) system. Animal experiments were conducted with the approval by the Animal Care Committee of the Shanghai Cancer Institute and in compliance with the National Institutes of Health Guide for the Care and Use of Laboratory Animals institutional guidelines.

### Plasmids and Lentivirus Packaging

GV492-BAP31 plasmid, short hairpin RNAs (shRNAs) targeting BAP31 (shBAP31) and the negative control (NC) were purchased from GeneChem (Shanghai, China). The short hairpin RNAs (shRNAs) targeting Snail (shSnail) and the plKO.1 vector were purchased from Horizon Discovery (Cambridge, United Kingdom). All plasmids were sequenced to blast with the information provided by NCBI or the companies above. Lentiviruses were packaged according to the protocol provided by Polyplus-transfection (Illkirch, France). The targeting sequences of shRNA were provided in Supplementary Table 1.

### RNA Extraction and Quantitative Real-Time Polymerase Chain Reaction

Total RNA was extracted with TRIzol reagent (Invitrogen, Carlsbad, CA, United States) and reversely transcribed into cDNA using the PrimeScript^TM^ RT Reagent Kit (TaKaRa Biotechnology, Shimogyo-ku, Japan). According to instructions in the SYBR Premix Ex Taq (TaKaRa Biotechnology), real-time PCR was performed. GAPDH was used as endogenous control. The primers designed for the relevant genes were provided in Supplementary Table 2.

### Western Blot

Cells were collected with protein extraction reagent (Thermo Fisher Scientific, United States) to extract total proteins and determine concentrations in BCA reagent. According to the concentration, protein was equally loaded and separated by SDS-PAGE. Then the protein in gel was transferred to PVDF membrane (Millipore, United States). The membrane was blocked with skim milk and incubated with corresponding primary antibody and HRP-conjugated secondary antibody. β-actin was used as endogenous control. Information on the antibodies was listed in Supplementary Table 3.

### Transwell Migration and Invasion Assays

Transwell migration and invasion assays were performed according to previous work (Song et al., 2019). Appropriate number of cells suspended by 200 μl serum-free medium were seeded into the upper chamber of the transwell (8 μm, Corning, United States), and 600 μl fresh DMEM with 10% FBS were added into the bottom of 24-well plates. In cell invasion experiments, transwell membranes were coated with matrigel while there were not in migration assays. After incubation at 37°C for corresponding time, cells were fixed in formaldehyde and stained by crystal violet staining for 10 min. Cells were captured and counted in three randomly chosen visual fields. Each experiment was performed in triplicate.

### Wound Scratch Assay

Cell migration was assessed by wound scratch assay. Firstly, we seeded HCC cells with BAP31 over-expression or knockdown into 6-well plate, and a 200-μl pipette tip was used to scratch on middle of the plate after cells grew to 80–90% confluence. Then, we washed the dish with PBS and replaced with medium in no FBS. At the indicated time points (0, 24, and 48 h), wound scratches at specific sites were captured in the microscope. Lastly, we measured the width at different timepoints and conducted the statistical analysis between groups. Each experiment was performed in triplicate.

### Immunofluorescent Assay

The cells were seeded onto chamber slide (Thermo Scientific, Waltham, MA, United States) for 24 h and fixed in 4% paraformaldehyde for 30 min. The slides were incubated with primary antibody in blocking solution overnight at 4°C. After being washed, the slides were incubated in Alexa 568-conjugated secondary antibody for 40 min and 4′6-diamino- 2-phenylindole in blocking solution (DAPI) for 20 min. Images were obtained with Laser Scanning Confocal Microscope (Leica TCS SP8, Germany).

### Statistical Analysis

All data were presented as the mean ± SD. Comparisons between two groups were performed by student’s *t*-test *via* GraphPad Prism 7 (GraphPad Software, La Jolla, CA, United States). Comparisons among three or more group comparisons were conducted using one-way ANOVA. A survival analysis was performed using Kaplan–Meier method. A bivariate correlation analysis was performed using the Pearson’s correlation method. *P*-value of ns (not significant) >0.05, ^∗^<0.05, ^∗∗^<0.01, ^∗^ and ^∗∗^ were considered significant.

## Results

### BAP31 Is Up-Regulated in HCC Tissues and Correlates With Poor Prognosis

**FIGURE 1:**
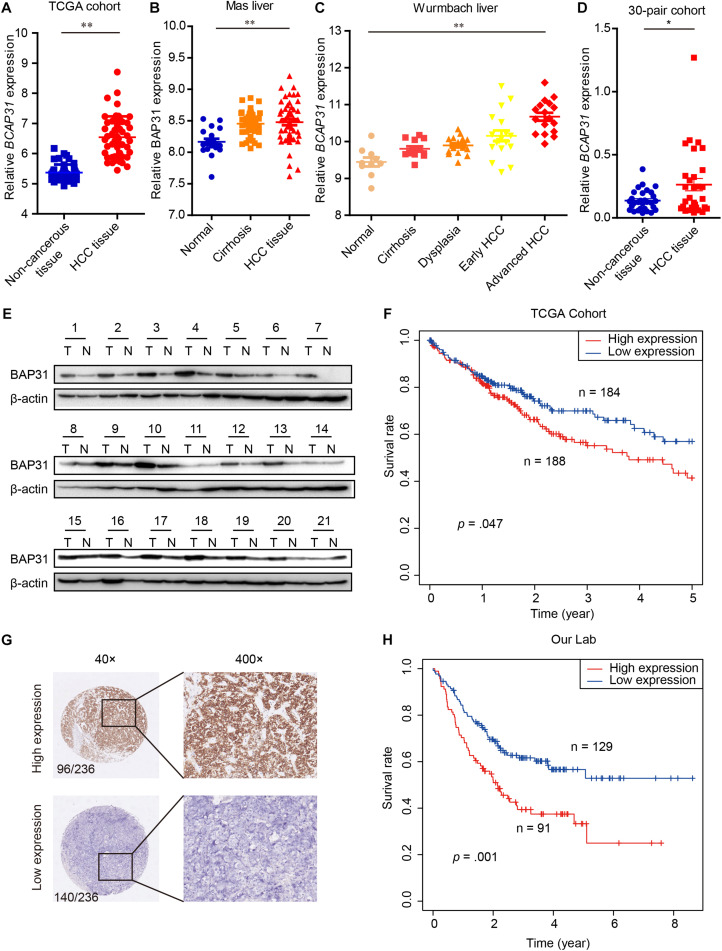
Elevated BAP31 expression indicated a poor prognosis in human HCC. **(A)** Relative BAP31 mRNA (*BCAP31*) expression in 50 paired HCC and adjacent non-cancerous tissues from TCGA. **(B)** Relative BAP31 mRNA expression in 19 normal liver tissues, 58 cirrhosis tissues and 38 HCC tissues from Mas liver cohort in GEO database. **(C)** Relative BAP31 mRNA expression in 10 normal liver tissues, 13 cirrhosis tissues, 17 dysplasia tissues, 18 early HCC tissues and 17 advanced HCC tissues from Wurmbach liver cohort in GEO database. **(D)** Relative BAP31 mRNA expression in 30 paired HCC and adjacent non-cancerous tissues from our lab. **(E)** BAP31 protein expression in 21 paired HCC and adjacent non-cancerous tissues from our lab, and β-actin was used as endogenous control. **(F)** Kaplan-Meier analysis of the relation of BAP31 mRNA expression with 5-year survival in TCGA cohort. **(G)** Representative images of IHC staining of BAP31 in human HCC tissues. (left) original magnification 40 ×, (right) original magnification 400 ×. **(H)** Kaplan-Meier analysis of the relation of BAP31 protein expression with overall survival in 236-patient cohort from our lab (^∗^*p* < 0.05, ^∗∗^*p* < 0.01).

EMT process process has been considered as main feature in tumor malignant transformation, which gets induced by TGF-β signaling (Giannelli et al., 2016). In this study, we treated cells with TGF-β and found an increased expression of BAP31 in tumor cells (Supplementary Figure 1). To explore the role of BAP31 in HCC, we first examined its mRNA (*BCAP31*) expression levels in TCGA cohort that the expression level was significantly higher in HCC tissues compared with non-cancerous liver tissues (Figure 1A). GEO database, including Mas liver and Wurmbach liver cohort, were analyzed that *BCAP31* expression elevated as malignant progression went along (Figures 1B,C). The human primary HCC tissues and non-cancerous liver tissues in our lab were detected, which showed an elevated *BCAP31* level in HCC tissues by qPCR (Figure 1D). Western blot was performed in 21-pair HCC and non-cancerous tissues that BAP31 protein expression was increased in HCC tissues at a ratio of 17/21 cases (80.95%) (Figure 1E and Supplementary Figure 2A). The TCGA cohort also indicated a lower methylated state of BAP31 promoter in HCC tissues, which might account for its increased transcription level (Supplementary Figure 2B). The pan-cancer view was analyzed in TCGA database that BAP31 level was also elevated in COAD (Colon adenocarcinoma), PAAD (Pancreatic adenocarcinoma), READ (Rectum adenocarcinoma) and THYM (Thymoma) (Supplementary Figure 2C).

The Kaplan–Meier analysis in TCGA cohort indicated that high BAP31 expression was positively associated with a shorter 5-year survival in HCC patients (Figure 1F, *p* = 0.047). According to immunohistochemistry staining score, 236 HCC patients were divided into high (96 cases) and low (140 cases) BAP31 expression groups (Figure 1G). Also, HCC patients with high BAP31 level had a shorter survival time (Figure 1H, *p* = 0.001), which was in accordance with the previous report (Zhang et al., 2020). Further analysis revealed that BAP31 expression positively correlated with tumor size (*p* = 0.030) and vascular invasion (*p* = 0.012, Table 1). Overall, these data suggested that BAP31 might serve as a predictive factor for HCC patients.

**TABLE 1:** Clinicopathological analysis of BAP31 expression in HCC tissues.

Clinicopathological Features	Number of cases	BAP31 expression		*p* value *p < 0.05
		Low	High	
**Gender**				
Male	190	110 (57.9%)	80 (42.1%)	0.364
Female	46	30 (65.2%)	16 (34.8%)	
**Age**				
≤ 50	121	68 (56.2%)	53 (43.8%)	0.404
> 50	114	72 (63.2%)	42 (36.8%)	
**Cirrhosis**				
Absent	38	19 (50.0%)	19 (50.0%)	0.202
Present	198	121 (61.1%)	77 (38.9%)	
**Vascular invasion**				
Absent	160	104 (65.0%)	56 (35.0%)	0.030*
Present	16	6 (37.5%)	10 (62.5%)	
**Edmondson’s grade**				
I–II	119	66 (55.5%)	53 (44.5%)	0.223
III–IV	117	74 (63.2%)	43 (36.8%)	
**Tumor size (cm)**				
≤ 5	113	76 (67.3%)	37 (32.7%)	0.012*
>5	116	59 (50.9%)	57 (49.1%)	

### BAP31 Promotes HCC Metastasis *in vitro* and *in vivo*

**FIGURE 2:**
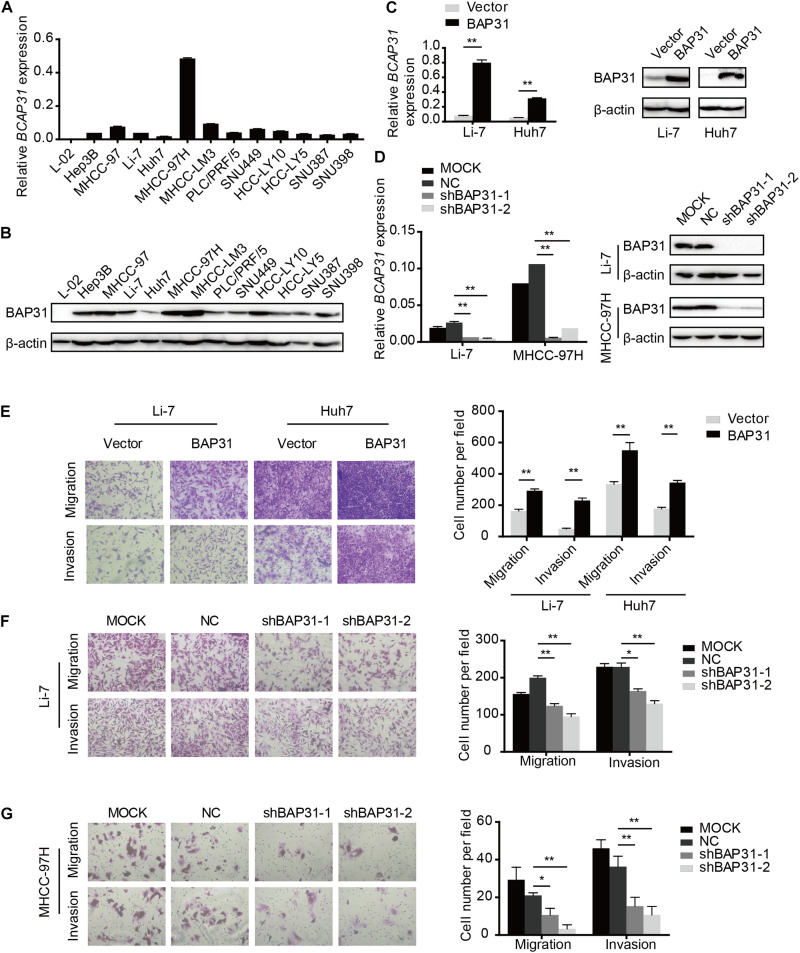
BAP31 facilitated HCC cell migration and invasion *in vitro*. **(A)** Relative mRNA expression level of BAP31 in normal liver cell L-02 and HCC cell lines. **(B)** Relative protein expression level of BAP31 in normal liver cell L-02 and HCC cell lines. **(C)** Relative mRNA and protein level of BAP31 in BAP31-overexpressed and vector control Li-7 and Huh7 cells. **(D)** Relative mRNA and protein level of BAP31 in Li-7 and MHCC-97H cell, which were divided into four groups: MOCK, NC, shBAP31-1, shBAP31-2. **(E)** Transwell assay analysis of the migratory and invasive abilities of HCC cells with BAP31 overexpression. Representative images (left) and the statistical analyses (right) were shown. **(F,G)** Transwell assay analysis of the migratory and invasive abilities of Li-7 and MHCC-97H cells with BAP31 knockdown. Representative images (left) and the statistical analyses (right) were shown. Results were presented as mean ± SD and *t*-test was conducted (*n* = 3, *p < 0.05, **p < 0.01).

To investigate the role of BAP31 in HCC, we examined BAP31 expression in normal liver cell L-02 and 12 HCC cell lines by qPCR and western blot (Figures 2A,B). Low BAP31 expression in L-02 cell was significantly increased in HCC cells, especially in MHCC-97H and MHCC-LM3 cells with high metastatic potential (Chakraborty et al., 2015; Chen et al., 2015). We stably over-expressed BAP31 in Li-7 and Huh7 cells with moderate or low BAP31 expression (Figure 2C), and knocked down BAP31 using shRNA in Li-7 and MHCC-97H cells (Figure 2D). Wound scratch assay showed that over-expression of BAP31 increased migratory abilities of Li-7 and Huh7 cells (Supplementary Figures 3A,B), and transwell migration and invasion assays indicated that over-expression of BAP31 promoted migration and invasion *in vitro* (Figure 2E). On the contrary, knockdown of BAP31 inhibited migratory and invasive abilities of Li-7 and MHCC-97H cells (Figures 2F,G and Supplementary Figures 4A,B).

**FIGURE 3:**
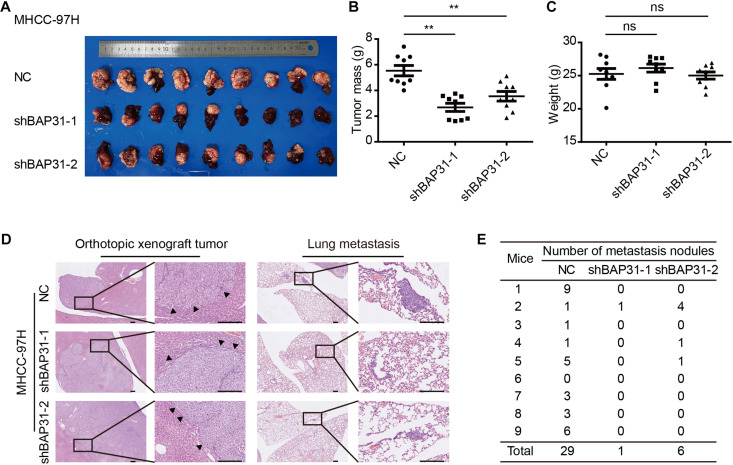
BAP31 promoted HCC progression *in vivo*. **(A–C)** Liver orthotopic xenograft experiments injected with shBAP31 and NC group MHCC-97H cells, including the representative images of liver tumors isolated from mice and quantifications of tumor weight and body weight. Results were presented as mean ± SD and *t*-test was conducted between NC and shBAP31-1/-2 group (ns *p* > 0.05, **p < 0.01). **(D,E)** H&E staining for liver and lung sections acquired from liver orthotopic mouse models, which included the representative images and statistics for the lung metastatic nodules. Scale bar: 200 μm. Arrows pointed to the margins between tumor and liver tissue.

To investigate *in vivo* function of BAP31 in the metastatic abilities, we orthotopically injected equal number of BAP31-silenced or control MHCC-97H cells in the liver of BALB/c nude mice. After 6 weeks the mice were sacrificed to weigh the tumor, of which the BAP31-silenced group gained less tumor weight than the control group while the body weight remained unaffected (Figures 3A–C). H&E staining for orthotopic tissues suggested that liver xenografts formed in control group tended to intersect with the normal liver regions while that in shBAP31 group had a relatively distinct margin, indicating a less invasive ability following BAP31 depletion (Figure 3D). Moreover, BAP31 availed the lung metastasis of HCC, with 8/9 mice exhibited lung metastasis in NC group while 1/9 and 3/9 mice for shBAP31-1/-2 group (Figures 3D,E). These findings indicated that BAP31 promoted HCC metastasis *in vitro* and *in vivo*.

### BAP31 Facilitates Snail-Mediated EMT Process

**FIGURE 4:**
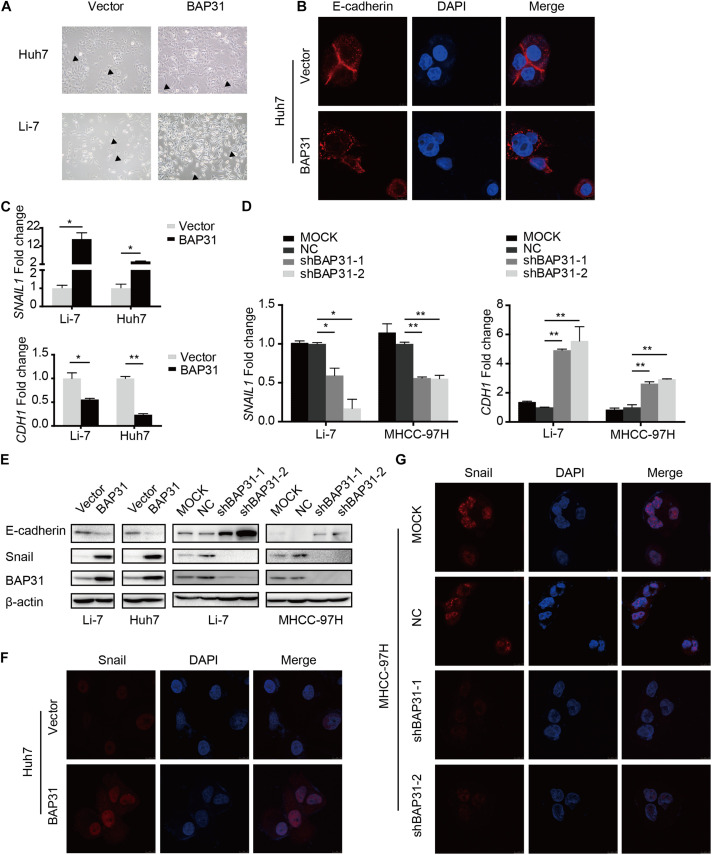
BAP31 induced Snail-mediated EMT process. **(A)** Morphological observations in Huh7 and Li-7 cells with BAP31 over-expression. Arrows pointed to the representative cell morphology. **(B)** Immunofluorescent (IF) assays were performed in Huh7 cells with BAP31 over-expression, which contained antibody against E-cadherin (red) and conjugated Alexa 568 secondary antibody, DAPI (blue) was used as the marker of nuclear. **(C)** Fold change of Snail (*SNAIL1*) and E-cadherin (*CDH1*) mRNA expression in Li-7 and Huh7 cells with BAP31 overexpression. Results were presented as mean ± SD and *t*-test was conducted between vector and BAP31 group (*n* = 3, *p < 0.05, **p < 0.01). **(D)** Fold change of Snail (*SNAIL1*) and E-cadherin (*CDH1*) mRNA expression in Li-7 and MHCC-97H cells, which were divided into four groups: MOCK, NC, shBAP31-1, shBAP31-2. Results were presented as mean ± SD and *t*-test was conducted between NC and shBAP31-1/-2 group (*n* = 3, *p < 0.05, **p < 0.01). **(E)** Western blot analysis showing protein expression of E-cadherin, Snail and BAP31 in HCC cells with over-expression and knockdown of BAP31. **(F)** Immunofluorescent (IF) assays were performed in Huh7 cell with BAP31 over-expression, which contained antibody against Snail (red) and conjugated Alexa 568 secondary antibody, DAPI (blue) was used as the marker of nuclear. The merge was referred to the overlay of Snail and DAPI. **(G)** Immunofluorescent (IF) assays were performed to test the nuclear contents of Snail in MHCC-97H cells with BAP31 knockdown.

The above results indicated that TGF-β-induced BAP31 promoted HCC metastasis. In transwell assay, it was observed that BAP31 attenuation partially reversed the promotional effects of TGF-β in cell migratory and invasive abilities (Supplementary Figure 5), implying that BAP31 might participate in TGF-β-induced EMT process. In BAP31-overexpressed cells, we observed a mesenchymal morphological transition compared to control group (Figure 4A). And the immunofluorescent assay (IF) showed that E-cadherin inclined to be weaker and dispersed in cell membrane upon BAP31 over-expression, therefore impairing the tight junction between cells (Figure 4B). The qPCR assay presented that Snail (*SNAIL1*) mRNA level increased in BAP31-overexpressed Li7 and Huh7 cells while E-cadherin (*CDH1*) mRNA level decreased compared to that in vector group (Figure 4C). On the contrary, attenuating BAP31 expression led to a lower expression level of *SNAIL1* along with a higher *CDH1* level (Figure 4D). Consistently, western blot and immunofluorescent assay (IF) showed that BAP31 over-expression enhanced the protein level of Snail (Figures 4E,F and Supplementary Figures 6A,C), while BAP31 knockdown reduced contents of Snail (Figures 4E,G and Supplementary Figures 6B,D). These findings implied that BAP31 increased expression of Snail and induced Snail-mediated EMT process.

### BAP31 Activates Snail-Mediated EMT *via* AKT/β-Catenin Pathway

**FIGURE 5:**
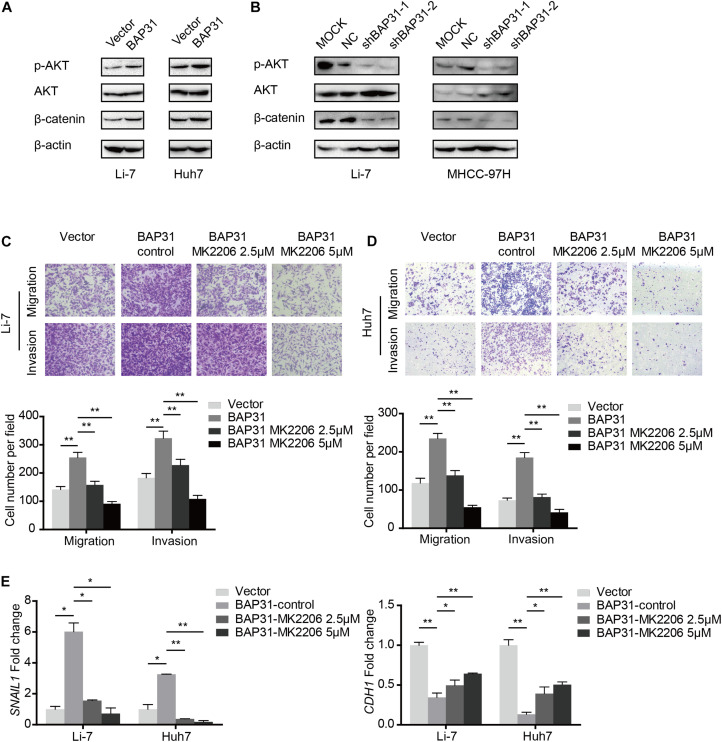
BAP31 promoted HCC progression *via* AKT/β-catenin/Snail pathway. **(A,B)** Western blot analysis showing protein expression of phosphorylated AKT, pan-AKT, β-catenin and BAP31 in HCC cells with overexpression and knockdown of BAP31. **(C,D)** Transwell assay analysis of the migratory and invasive abilities in Li-7 and Huh7 cells, which were divided into four groups: vector, BAP31 (DMSO-control), BAP31 (MK2206 2.5 μM) and BAP31 (MK2206 5 μM). **(E)** Fold change of Snail (*SNAIL1*) and E-cadherin (*CDH1*) mRNA expression in Li-7 and Huh7 cell, which were divided into four groups: vector, BAP31 (DMSO-control), BAP31 (MK2206 2.5 μM) and BAP31 (MK2206 5 μM). Results were presented as mean ± SD and *t*-test was conducted between vector and BAP31 (DMSO-control), BAP31 (DMSO-control) and BAP31 (MK2206 2.5 μM/5 μM) group (*n* = 3, *p < 0.05, **p < 0.01).

Since it has been elucidated that BAP31 increased Snail-mediated EMT process, we may wonder the way how it acted. In western blot, BAP31 over-expression elevated protein level of p-AKT and β-catenin, which were reduced upon BAP31 attenuation (Figures 5A,B). Then BAP31-overexpressed cells were treated with AKT inhibitor, MK-2206, and AKT inhibitor impaired the promotional effect of BAP31 on cell migration and invasion (Figures 5C,D). As it was investigated that AKT might act as upstream for Snail-mediated EMT process (Liu et al., 2014), we proceeded to further validate this. The qPCR results showed that AKT inhibitor was able to offset the increased Snail level, which was induced upon BAP31 over-expression (Figure 5E). Moreover, the decreased mRNA level of E-cadherin in BAP31-overexpressed cells was rescued by AKT inhibitor (Figure 5E). The above results indicated that BAP31 regulated Snail-mediated EMT *via* AKT/β-catenin pathway.

### BAP31 Promotes HCC Cell Metastasis *via* Snail

**FIGURE 6:**
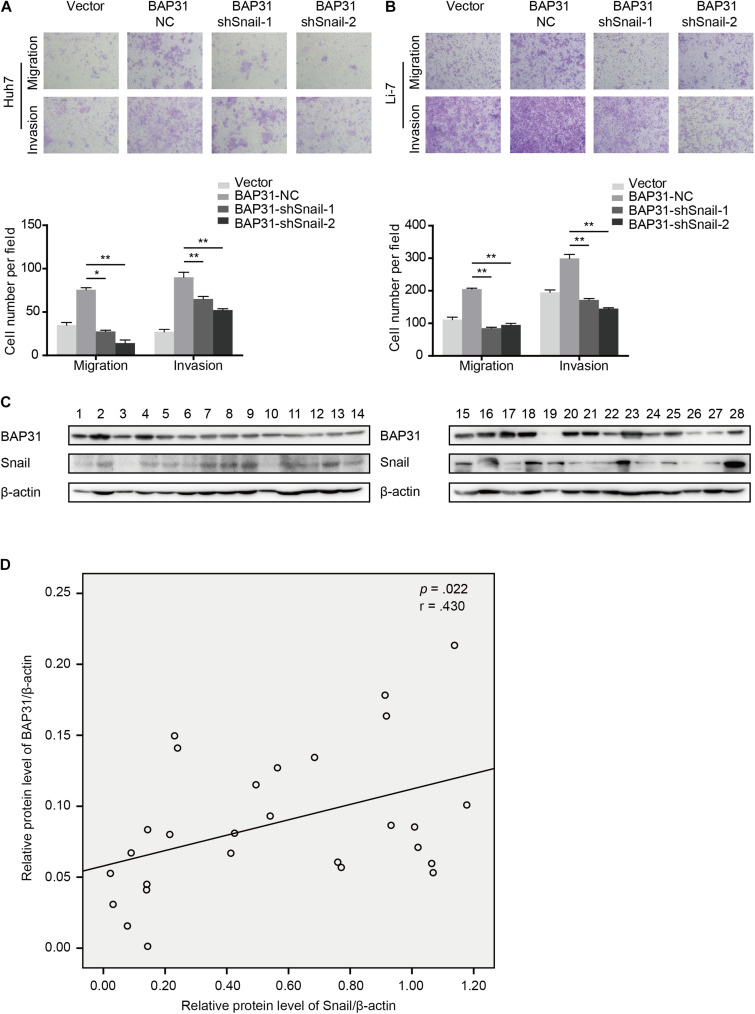
BAP31 promoted HCC cell migration and invasion *via* Snail and there was positive correlation between BAP31 and Snail in HCC. **(A–B)** Transwell assay analysis of the migratory and invasive abilities of Huh7 and Li-7 cells, which were divided into four groups: vector, BAP31-NC, BAP31-shSnail-1 and BAP31-shSnail-2. Representative images (upper) and the statistical analysis (lower) were shown. Results were presented as mean ± SD and *t*-test was conducted between BAP31-NC and BAP31-shSnail-1/-2 group (*n* = 3, *p < 0.05, **p < 0.01). **(C–D)** Western blot analysis showed the positive correlation between protein expression of BAP31 and Snail in HCC tissues (*n* = 28, *p* = 0.022, *r* = 0.430).

As a crucial regulator in EMT process, Snail possesses the ability to facilitate tumor progressive acquisition and promote tumor metastasis *via* repressing epithelial marker E-cadherin (Kudo-Saito et al., 2009). We further investigated whether BAP31 promoted HCC metastasis *via* Snail. In BAP31-overexpressed cells, Snail depletion tended to weaken the enhanced metastatic abilities compared to the vector group (Figures 6A,B). Moreover, western blot indicated that the expression level of BAP31 positively correlated with Snail in 28 HCC tissues (*p* = 0.022, *r* = 0.430, Figures 6C,D). In conclusion, BAP31 might promote HCC progression *via* Snail and there existed a co-expression relation between the two factors in HCC.

## Discussion

It has been well acknowledged that hepatocellular carcinoma is ranked as the sixth common neoplasm, leading third in the cancer-related death (Chen et al., 2016; Mak et al., 2020). Metastasis is considered as the primary cause of death in HCC patients, which may attribute to its high malignancy and heterogeneity (Marquardt et al., 2015). Hence, it is urgent to identify novel factors involved in aggressive transformation and metastasis, so as to better understand molecular mechanism in HCC progression. As a cancer/testis antigen, BAP31 has been correlated with patient survival in multiple kinds of cancer (Dang et al., 2018; Xu et al., 2019). BAP31 was up-regulated in cervical cancer, malignant melanomas and lung carcinomas and its high expression indicated a poor prognosis (Yu et al., 2015; Seo et al., 2017; Dang et al., 2018). In the present study, we have elucidated that BAP31 was up-regulated in HCC compared to normal tissues and high BAP31 expression contributed to poor prognosis, which suggested BAP31 might serve as a potential prognostic factor in HCC. In HCC patients after surgical resection, low expression levels of BAP31 might indicate poor outcome (Tan et al., 2016), which was contradictory with our samples and one recent study (Zhang et al., 2020). However, patients included in our analysis received no preoperative radiation or chemotherapy, which differed with the samples they collected and subsequently led to a different result. As cancer progression is a complex and heterogeneous process, it still needs precisely molecular classification for HCC evaluation.

There are reports that BAP31 promotes proliferation and metastasis in multiple cancers (Dang et al., 2018; Wang et al., 2019, 2020; Xu et al., 2019). In the current study, we have initially illustrated that BAP31 promoted HCC migration, invasion and metastasis both *in vitro* and *in vivo.* Ectopic expression of BAP31 induced a more aggressive phenotype, with mesenchymal-like morphology and disruption of epithelial marker E-cadherin, and a more invasive boundary in liver xenografts established in mice compared to BAP31 depletion group. Moreover, TGF-β induced BAP31 expression so as to promote HCC metastasis. There are reports that BAP31 knockdown reduces expression level of TGF-β1, MMP-2, MMP-9, ROCK1, α-SMA, Vimentin and N-cadherin in cervical cancer (Wang et al., 2019), and BAP31 affects F-actin distribution to promote migration and invasion of lung cancer cells (Wang et al., 2020). Recent study also suggests that miR-362 can inhibit tumor progression *via* BAP31 and TGF-β/Smad pathway in cervical cancer (Yang et al., 2021). These results indicate that BAP31 may participate in mesenchymal phenotype switch in cancer progression and facilitate metastasis.

EMT process has long drawn attention in its regulation in tumor progression, involving a variety of factors such as TGF-β1, Snail, Slug, Cadherins and so on (Giannelli et al., 2016; Losic et al., 2020). This study implied that BAP31 tended to increase expression of Snail and both factors were positively correlated in HCC, suggesting that BAP31 may facilitate HCC metastasis *via* Snail-mediated EMT induction. As a crucial transcription factor in EMT process, Snail regulates downstream factors such as E-cadherin and facilitates cancer metastasis (Peinado et al., 2007). This work also elucidated that Snail partially mediated the pro-metastatic role of BAP31. In accordance with the research in human embryonic stem cell (Kim et al., 2014), AKT/β-catenin pathway was activated under BAP31 over-expression so as to promote HCC progression in this research. It has been reported that AKT/β-catenin induces Snail-mediated EMT process (Liu et al., 2014), giving us a hint that BAP31 might stimulate Snail-mediated EMT process *via* AKT/β-catenin activation. While there has been complex interaction between AKT activation and TGF-β-induced EMT (Karimi Roshan et al., 2019), it needs further investigation whether BAP31 directly or indirectly interacts with other proteins to exert its oncogenic roles in this regulatory network.

In summary, it has been demonstrated that BAP31 promotes HCC metastasis through AKT/β-catenin and Snail-mediated EMT pathway. High BAP31 level indicates a poor prognosis in HCC patients, which may serve as a potential biomarker. Our findings highlight the role and molecular mechanism of BAP31 in HCC progression and provide valuable information for clinical practices.

## Data Availability Statement

The original contributions presented in the study are included in the article/Supplementary Material, further inquiries can be directed to the corresponding author/s.

## Ethics Statement

The studies involving human participants were reviewed and approved by Research Ethics Committee of Renji Hospital, Shanghai Jiao Tong University School of Medicine. The patients/participants provided their written informed consent to participate in this study. The animal study was reviewed and approved by Animal Care Committee of the Shanghai Cancer Institute.

## Author Contributions

TL, HH, and HL conceived and designed experiments. TL, JY, CG, FZ, CM, WJ, YS, QG, and MY conducted the relevant experiments. TL and JY analyzed the data and wrote the manuscript. TC, HX, and YC collected tissue samples. JL, HH, and HL provided critical comments, suggestions, and revised the manuscript. All authors read and approved the final manuscript.

## Conflict of Interest

The authors declare that the research was conducted in the absence of any commercial or financial relationships that could be construed as a potential conflict of interest.
