# Supplementary material for: B-Cell Receptor-Associated Protein 31 Promotes Metastasis via AKT/β-Catenin/Snail Pathway in Hepatocellular Carcinoma
Source: Front Mol Biosci. 2021 Jun 11;8:656151. doi: 10.3389/fmolb.2021.656151 (PMC8231437; doi:10.3389/fmolb.2021.656151)
Supplement: Supplementary file 1 [file Table_1.docx]

B-cell receptor-associated protein 31 promotes metastasis via AKT/β-catenin/Snail pathway in hepatocellular carcinoma

**Tengfei Liu^1†^, Junming Yu^1†^, Chao Ge^1^, Fangyu Zhao^1^, Chunxiao Miao^1^, Wenjiao Jin^1^, Yang Su^1^, Qin Geng^1^, Taoyang Chen^2^, Haiyang Xie^3^, Ying Cui^4^, Ming Yao^1^, Jinjun Li^1^, Helei Hou^5*^, Hong Li^1,5*^**

^1 State Key Laboratory of Oncogenes and Related Genes, Shanghai Cancer Institute, Renji Hospital, Shanghai Jiao Tong University School of Medicine, Shanghai, China^

^2 Qi Dong Liver Cancer Institute, Qi Dong, China^

^3 Department of General Surgery, the First Affiliated Hospital, School of Medicine, Zhejiang University, Hangzhou, China^

^4 Cancer Institute of Guangxi, Nanning, China^

^5 Precision Medicine Center of Oncology, the Affiliated Hospital of Qingdao University, Qingdao, China^

^† These authors have contributed equally to this work and share first authorship.^

*** Correspondence:**Helei Hou, Email: houhelei@qdu.edu.cn

Hong Li, Email: [hongli@shsci.org](mailto:hongli@shsci.org)


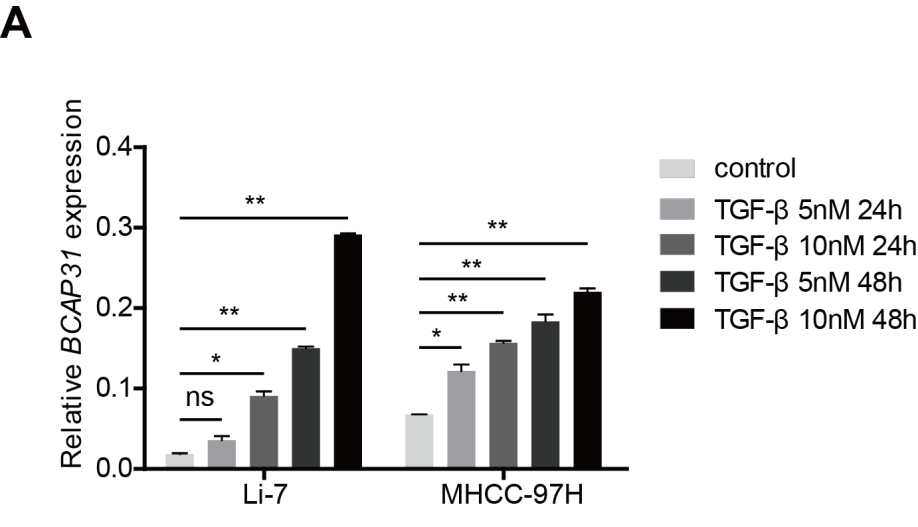


**Figure S1 | TGF-β induced BAP31 expression in HCC cells. (A)** Relative BAP31 mRNA expression in HCC cells under the treatment of TGF-β. Results were presented as mean ± SD and t-test was conducted between control and TGF-β treatment group (n = 3, ns = no significance, **p* < 0.05, ***p* < 0.01).


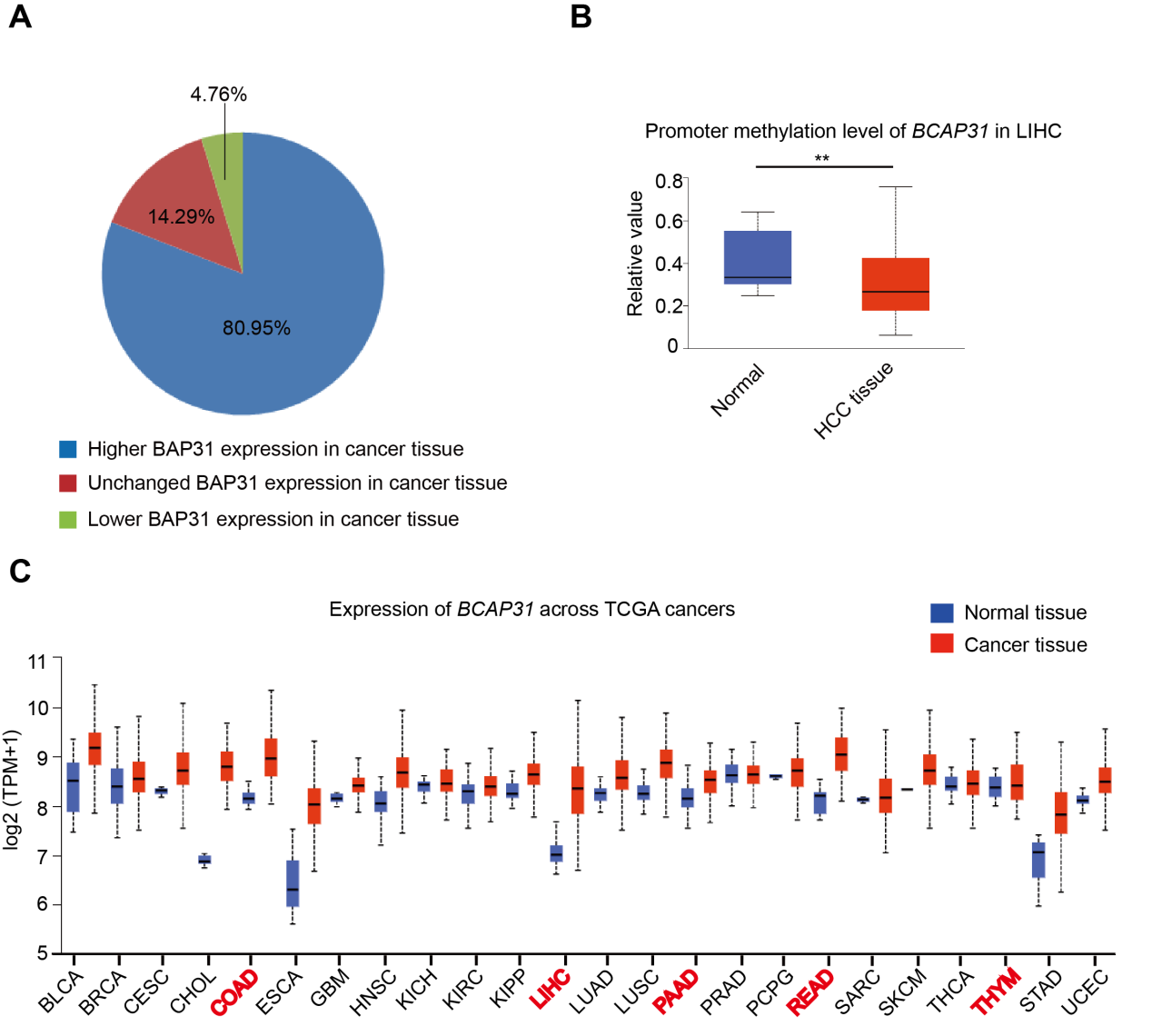


**Figure S2 | The expression level and methylation level of BAP31 in cancer. (A)** Statistical analysis of western blot in 21-pair HCC and non-cancerous tissues: 80.95% cases with BAP31 expression higher in cancer tissues, 14.29% cases with BAP31 expression unchanged and 4.76% cases lower in cancer tissues. **(B)** The promoter methylation level of BAP31 in TCGA LIHC cohort. **(C)** TCGA pan-cancer view of BAP31 expression. Red fonts referred to higher BAP31 expression in cancer tissues. BLCA: Bladder urothelial carcinoma, BRCA: Breast invasive carcinoma, CESC: Cervical squamous cell carcinoma and endocervical adenocarcinoma, CHOL: Cholangio carcinoma, COAD: Colon adenocarcinoma, ESCA: Esophageal carcinoma, GBM: Glioblastoma multiforme, HNSC: Head and Neck squamous cell carcinoma, KICH: Kidney chromophobe, KIRC: Kidney renal clear cell carcinoma, KIRP: Kidney renal papillary cell carcinoma, LIHC: Liver hepatocellular carcinoma, LUAD: Lung adenocarcinoma, LUSC: Lung squamous cell carcinoma, PAAD: Pancreatic adenocarcinoma, PRAD: Prostate adenocarcinoma, PCPG: Pheochromocytoma and Paraganglioma, READ: Rectum adenocarcinoma, SARC: Sarcoma, SKCM: Skin cutaneous melanoma, THCA: Thyroid carcinoma, THYM: Thymoma, STAD: Stomach adenocarcinoma, UCEC: Uterine corpus endometrial carcinoma. Results were presented as mean ± SD and t-test was conducted (***p* < 0.01).


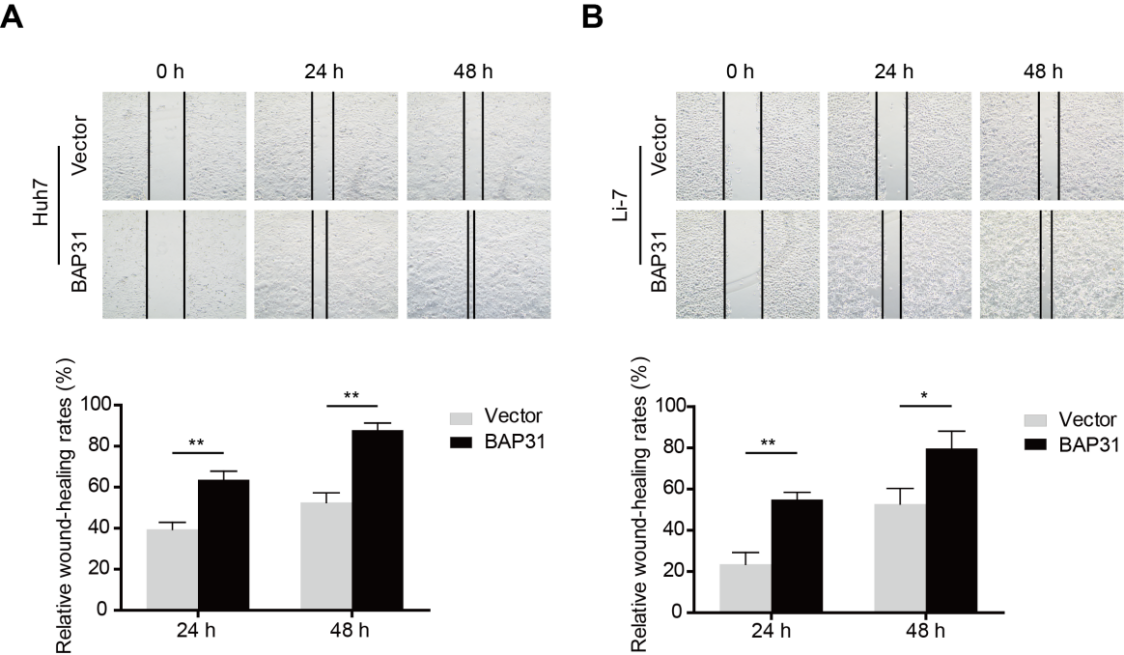


**Figure S3 | BAP31 overexpression increased the wound-healing abilities of HCC cells. (A, B)** Wound scratched assay of Huh7 and Li-7 cell with BAP31 overexpression. Relative wound-healing rate = (width at 0 h - width at 24 h/48 h)/width at 0 h * 100%. Results were presented as mean ± SD and t-test was conducted between vector and BAP31 group (n = 3, **p* < 0.05, ***p* < 0.01).

**
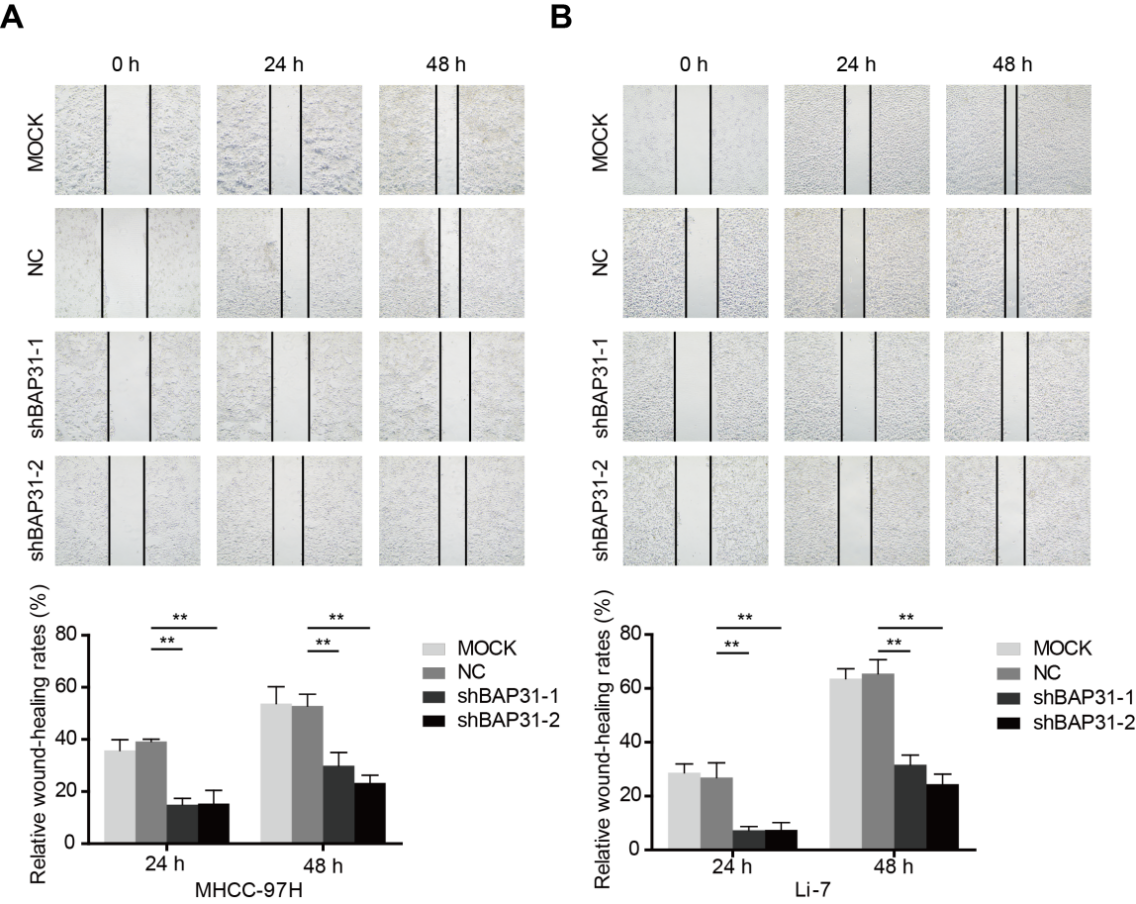
**

**Figure S4 |** **BAP31 knockdown decreased the wound-healing abilities of HCC cells. (A, B)** Wound scratched assay of MHCC-97H and Li-7 cell with BAP31 knockdown. Relative wound-healing rate = (width at 0 h - width at 24 h/48 h)/width at 0 h * 100%. Results were presented as mean ± SD and t-test was conducted between NC and shBAP31-1/-2 group (n = 3, ***p* < 0.01).


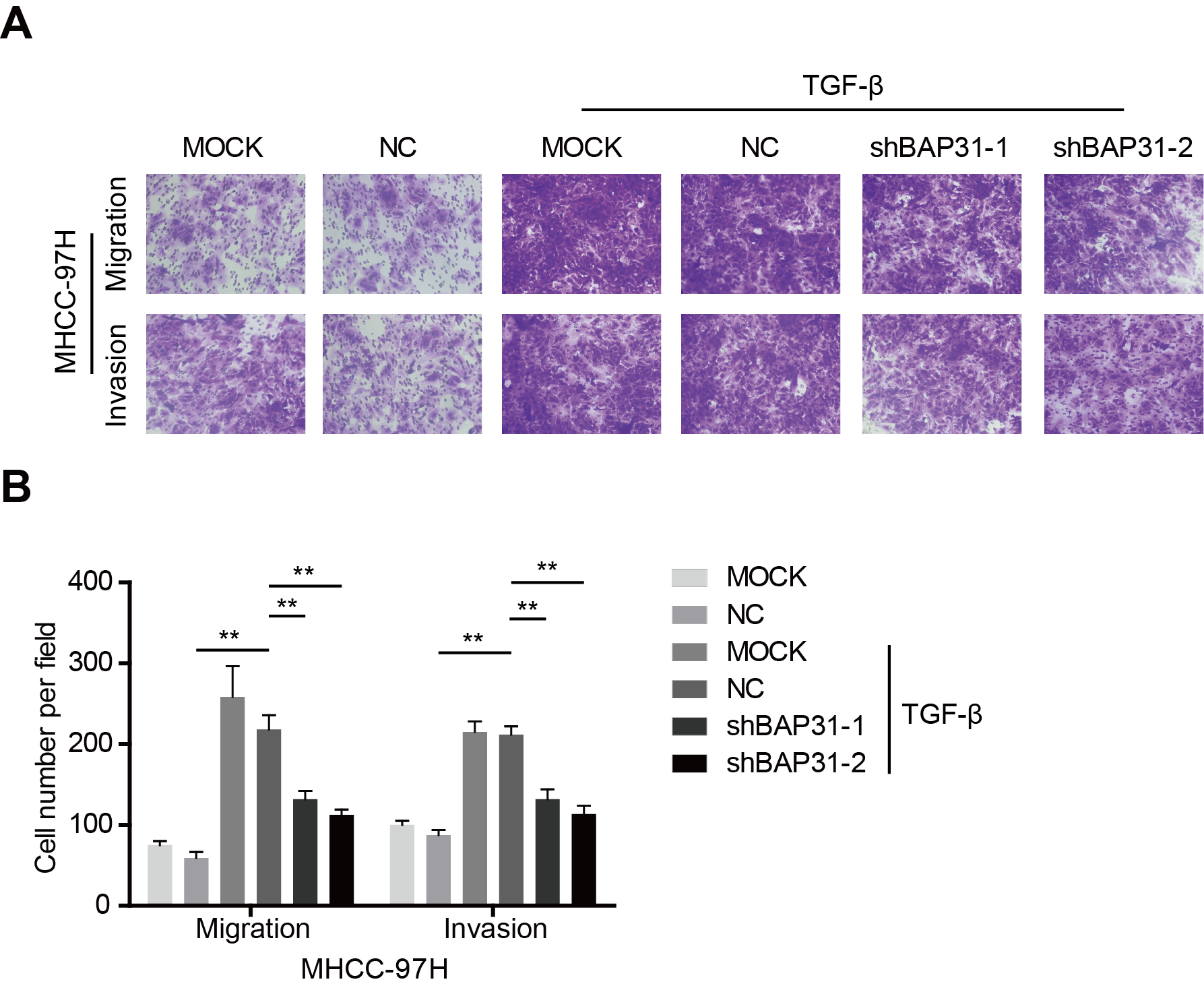


**Figure S5 | TGF-β induced BAP31 to facilitate migration and invasion in HCC cells. (A)** Transwell assay of the migratory and invasive abilities of HCC cells, which were divided into six groups: MOCK, NC, TGF-β-MOCK, TGF-β-NC, TGF-β-shBAP31-1 and TGF-β-shBAP31-2. **(B)** Statistical analysis of transwell assay. Results were presented as mean ± SD and t-test was conducted between NC and TGF-β-NC, TGF-β-NC and TGF-β-shBAP31-1/-2 (n = 3, ***p* < 0.01).


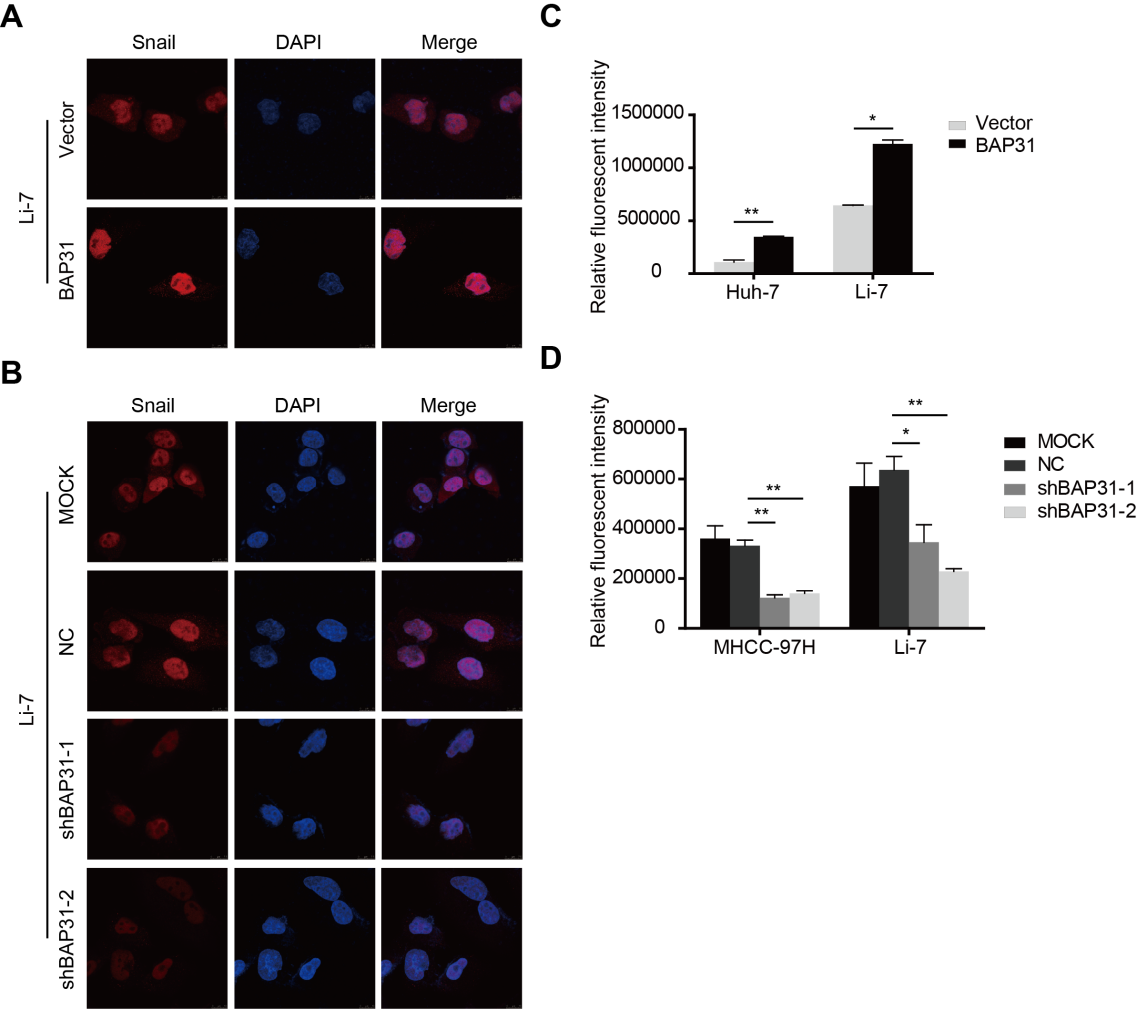


**Figure S6 | BAP31 increased the nuclear contents of Snail. (A, B)** Immunofluorescent (IF) assays were performed in Li-7 cells with BAP31 overexpression or knockdown, which contained antibody against Snail (red) and conjugated Alexa 568 secondary antibody, DAPI (blue) was used as the marker of nuclear. The merge was referred to the overlay of Snail and DAPI. **(C)** Quantifications of fluorescent intensity of Snail in Huh7 and Li-7 cells with BAP31 overexpression. Results were presented as mean ± SD and t-test was conducted between vector and BAP31 group (n = 3, **p* < 0.05, ***p* < 0.01). **(D)** Quantifications of fluorescent intensity of Snail in MHCC-97H and Li-7 cells with BAP31 knockdown. Results were presented as mean ± SD and t-test was conducted between NC and shBAP31-1/-2 group (n = 3, **p* < 0.05, ***p* < 0.01).

**Table S1 | The target sequences for shRNA**

| Name | Target Sequence (5’-3’) |
| --- | --- |
| NC | TTCTCCGAACGTGTCACGT |
| *BCAP31*-shRNA-1 | CGCCTGGTGACTCTCATTT |
| *BCAP31*-shRNA-2 | CGATGCCGTGCGCGAAATT |
| *SNAI1*-shRNA-1 | CCAGGCTCGAAAGGCCTTCAA |
| *SNAI1*-shRNA-2 | CCAATCGGAAGCCTAACTACA |

**Table S2 | The primers used for qRT-PCR**

| Primer Name | Primer Sequence (5’-3’) |
| --- | --- |
| H-*GAPDH*-F  H-*GAPDH*-R | AGAAGGCTGGGGCTCATTTG  AGGGGCCATCCACAGTCTTC |
| H-*BCAP31*-F | CTGCTGTCCTTCCTGCTTAG |
| H-*BCAP31*-R | CTCCTGTTCTCTTCCTCCAAC |
| H-*SNAIL1*-F | ATCGGAAGCCTAACTACAGCG |
| H-*SNAIL1*-R | TCGGATGTGCATCTTGAGGG |
| H-*CDH1*-F | ATTCTGATTCTGCTGCTCTTG |
| H-*CDH1*-R | AGTCCTGGTCCTCTTCTCC |

**Table S3 | The antibodies used in this study**

| Antibody | Host | Catalogue | Dilution | Application | | Corporation | |
| --- | --- | --- | --- | --- | --- | --- | --- |
| **Primary antibodies** | | | | | | | |
| BAP31 | Mouse IgG | sc393810 | 1:500/1:200 | WB/IHC | Santa Cruz | | |
| E-cadherin | Rabbit IgG | 3195 | 1:500/1:50 | WB/IF | Cell signaling | | |
| Snail | Mouse IgG | 3895 | 1:500/1:50 | WB/IF | Cell signaling | | |
| p-AKT | Rabbit IgG | 4060 | 1:1000 | WB | Cell signaling | | |
| pan-AKT | Rabbit IgG | 4691 | 1:1000 | WB | Cell signaling | | |
| β-catenin | Mouse IgG | 610153 | 1:1000 | WB | BD Biosciences | | |
| β-actin | Mouse IgG | ab49900 | 1:10000 | WB | Abcam | | |
| **Secondary antibodies** | | | | | | | |
| HRP-anti-  Mouse IgG | Goat | A0545 | 1:4000 | WB | Sigma-Aldrich | | |
| HRP-anti-  Rabbit IgG | Goat | sc2060 | 1:4000 | WB | Santa Cruz | | |
| EnViSion | Detection | Kit K5007 |  | IHC | DAKO | | |
| (Peroxidase/DAB; Rabbit) | | | | | | | |
